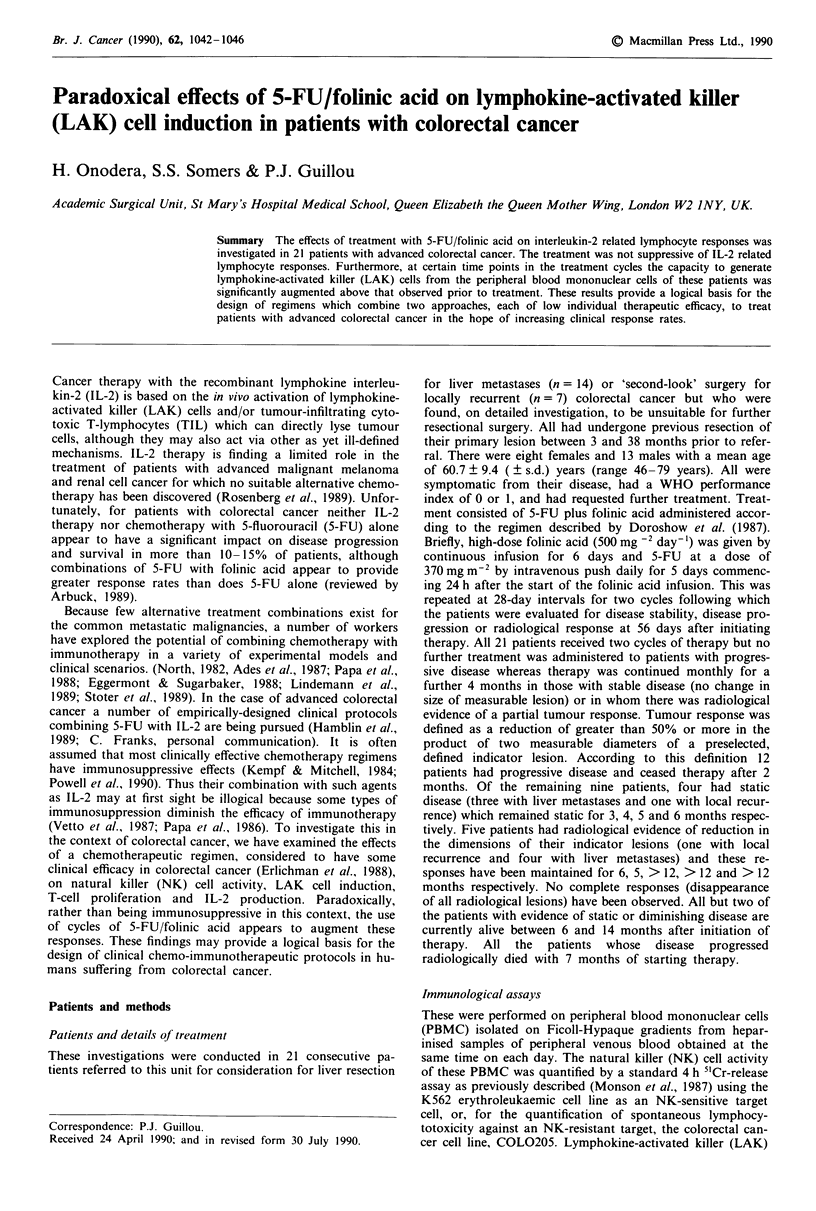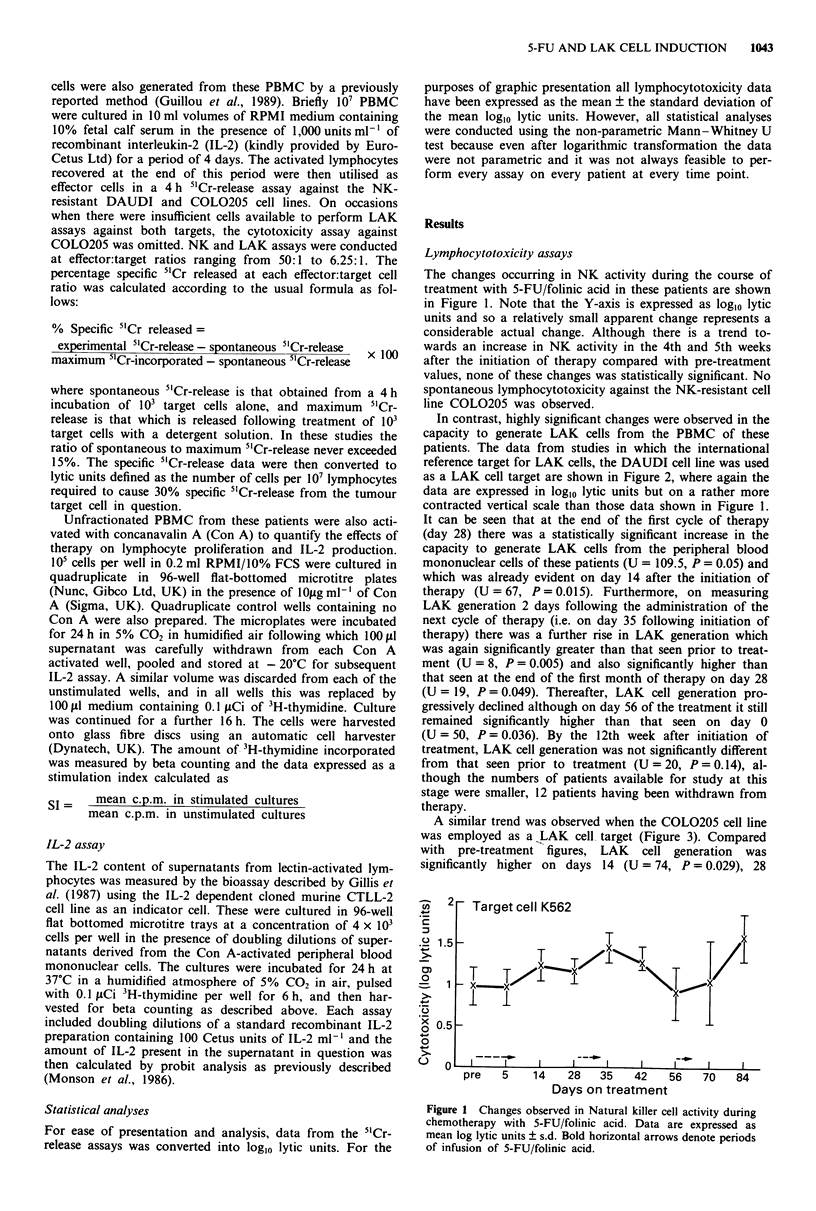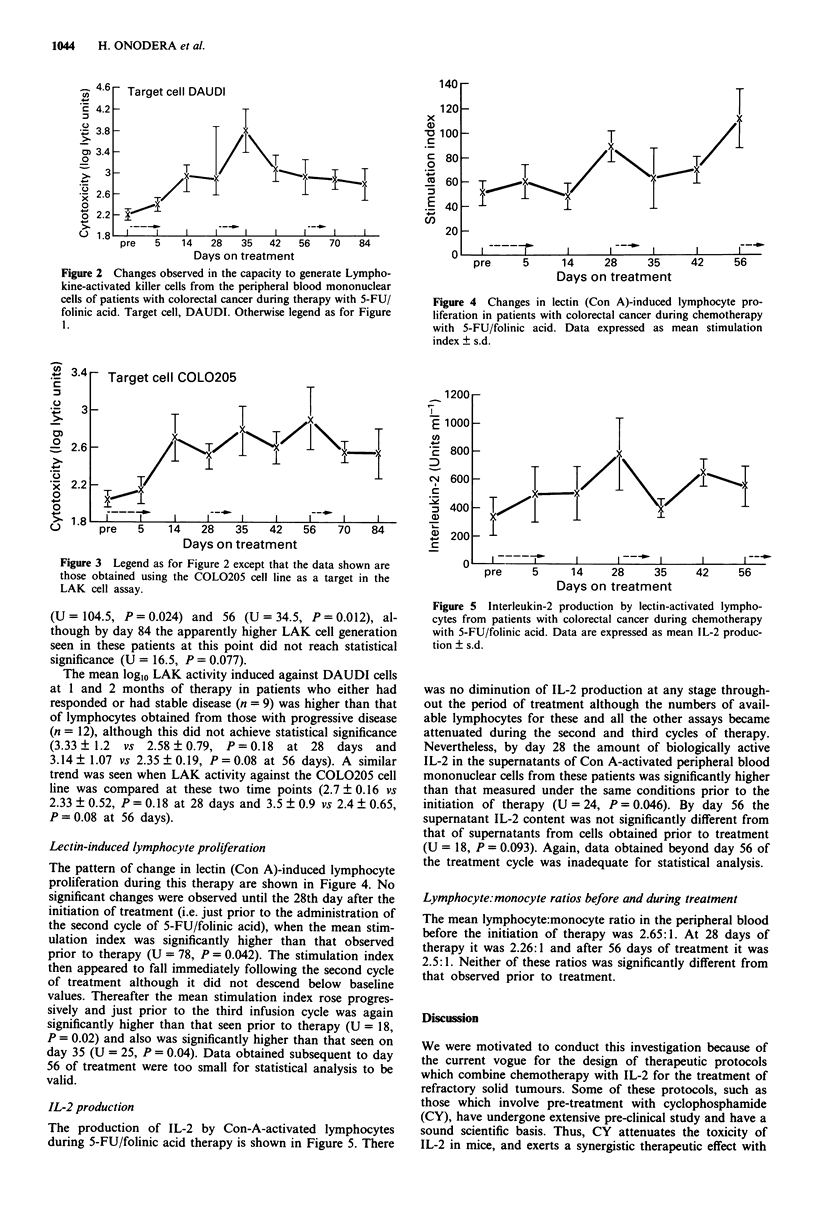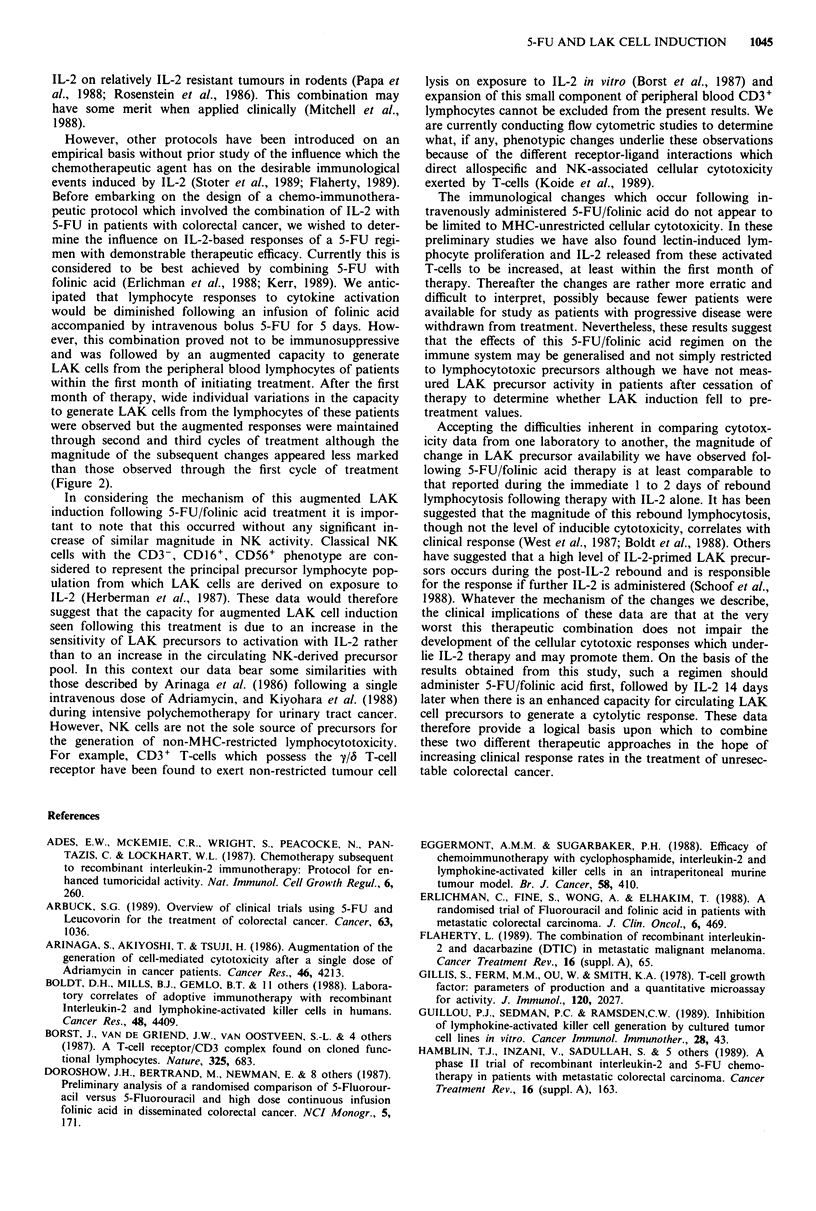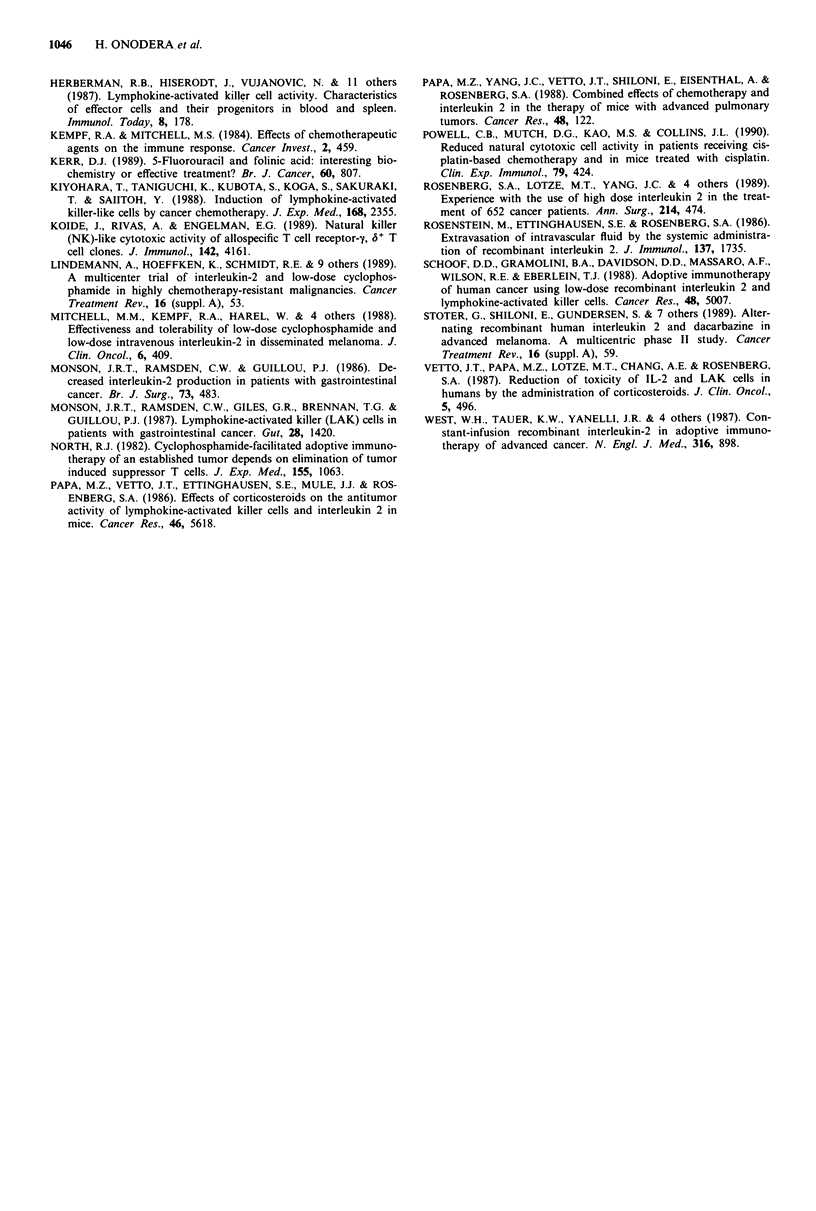# Paradoxical effects of 5-FU/folinic acid on lymphokine-activated killer (LAK) cell induction in patients with colorectal cancer.

**DOI:** 10.1038/bjc.1990.436

**Published:** 1990-12

**Authors:** H. Onodera, S. S. Somers, P. J. Guillou

**Affiliations:** Academic Surgical Unit, St Mary's Hospital Medical School, London, UK.

## Abstract

The effects of treatment with 5-FU/folinic acid on interleukin-2 related lymphocyte responses was investigated in 21 patients with advanced colorectal cancer. The treatment was not suppressive of IL-2 related lymphocyte responses. Furthermore, at certain time points in the treatment cycles the capacity to generate lymphokine-activated killer (LAK) cells from the peripheral blood mononuclear cells of these patients was significantly augmented above that observed prior to treatment. These results provide a logical basis for the design of regimens which combine two approaches, each of low individual therapeutic efficacy, to treat patients with advanced colorectal cancer in the hope of increasing clinical response rates.


Br. J. Cancer (1990), 62, 1042–1046 © Macmillan Press Ltd., 1990

### Paradoxical effects of 5-FU/folinic acid on lymphokine-activated killer (LAK) cell induction in patients with colorectal cancer

H. Onodera, S.S. Somers & P.J. Guillou

*Academic Surgical Unit, St Mary's Hospital Medical School, Queen Elizabeth the Queen Mother Wing, London W2 1NY, UK.*

**Summary** The effects of treatment with 5-FU/folinic acid on interleukin-2 related lymphocyte responses was investigated in 21 patients with advanced colorectal cancer. The treatment was not suppressive of IL-2 related lymphocyte responses. Furthermore, at certain time points in the treatment cycles the capacity to generate lymphokine-activated killer (LAK) cells from the peripheral blood mononuclear cells of these patients was significantly augmented above that observed prior to treatment. These results provide a logical basis for the design of regimens which combine two approaches, each of low individual therapeutic efficacy, to treat patients with advanced colorectal cancer in the hope of increasing clinical response rates.

Cancer therapy with the recombinant lymphokine interleukin-2 (IL-2) is based on the *in vivo* activation of lymphokine-activated killer (LAK) cells and/or tumour-infiltrating cytotoxic T-lymphocytes (TIL) which can directly lyse tumour cells, although they may also act via other as yet ill-defined mechanisms. IL-2 therapy is finding a limited role in the treatment of patients with advanced malignant melanoma and renal cell cancer for which no suitable alternative chemotherapy has been discovered (Rosenberg *et al.*, 1989). Unfortunately, for patients with colorectal cancer neither IL-2 therapy nor chemotherapy with 5-fluorouracil (5-FU) alone appear to have a significant impact on disease progression and survival in more than 10–15% of patients, although combinations of 5-FU with folinic acid appear to provide greater response rates than does 5-FU alone (reviewed by Arbuck, 1989).

Because few alternative treatment combinations exist for the common metastatic malignancies, a number of workers have explored the potential of combining chemotherapy with immunotherapy in a variety of experimental models and clinical scenarios. (North, 1982, Ades *et al.*, 1987; Papa *et al.*, 1988; Eggermont & Sugarbaker, 1988; Lindemann *et al.*, 1989; Stoter *et al.*, 1989). In the case of advanced colorectal cancer a number of empirically-designed clinical protocols combining 5-FU with IL-2 are being pursued (Hamblin *et al.*, 1989; C. Franks, personal communication). It is often assumed that most clinically effective chemotherapy regimens have immunosuppressive effects (Kempf & Mitchell, 1984; Powell *et al.*, 1990). Thus their combination with such agents as IL-2 may at first sight be illogical because some types of immunosuppression diminish the efficacy of immunotherapy (Vetto *et al.*, 1987; Papa *et al.*, 1986). To investigate this in the context of colorectal cancer, we have examined the effects of a chemotherapeutic regimen, considered to have some clinical efficacy in colorectal cancer (Erlichman *et al.*, 1988), on natural killer (NK) cell activity, LAK cell induction, T-cell proliferation and IL-2 production. Paradoxically, rather than being immunosuppressive in this context, the use of cycles of 5-FU/folinic acid appears to augment these responses. These findings may provide a logical basis for the design of clinical chemo-immunotherapeutic protocols in humans suffering from colorectal cancer.

### Patients and methods

### *Patients and details of treatment*

These investigations were conducted in 21 consecutive patients referred to this unit for consideration for liver resection for liver metastases ($n = 14$) or 'second-look' surgery for locally recurrent ($n = 7$) colorectal cancer but who were found, on detailed investigation, to be unsuitable for further resectional surgery. All had undergone previous resection of their primary lesion between 3 and 38 months prior to referral. There were eight females and 13 males with a mean age of $60.7 \pm 9.4$ ($\pm$ s.d.) years (range 46–79 years). All were symptomatic from their disease, had a WHO performance index of 0 or 1, and had requested further treatment. Treatment consisted of 5-FU plus folinic acid administered according to the regimen described by Doroshow *et al.* (1987). Briefly, high-dose folinic acid (500 mg $^{-2}$ day$^{-1}$) was given by continuous infusion for 6 days and 5-FU at a dose of 370 mg m$^{-2}$ by intravenous push daily for 5 days commencing 24 h after the start of the folinic acid infusion. This was repeated at 28-day intervals for two cycles following which the patients were evaluated for disease stability, disease progression or radiological response at 56 days after initiating therapy. All 21 patients received two cycles of therapy but no further treatment was administered to patients with progressive disease whereas therapy was continued monthly for a further 4 months in those with stable disease (no change in size of measurable lesion) or in whom there was radiological evidence of a partial tumour response. Tumour response was defined as a reduction of greater than 50% or more in the product of two measurable diameters of a preselected, defined indicator lesion. According to this definition 12 patients had progressive disease and ceased therapy after 2 months. Of the remaining nine patients, four had static disease (three with liver metastases and one with local recurrence) which remained static for 3, 4, 5 and 6 months respectively. Five patients had radiological evidence of reduction in the dimensions of their indicator lesions (one with local recurrence and four with liver metastases) and these responses have been maintained for 6, 5, $>12$, $>12$ and $>12$ months respectively. No complete responses (disappearance of all radiological lesions) have been observed. All but two of the patients with evidence of static or diminishing disease are currently alive between 6 and 14 months after initiation of therapy. All the patients whose disease progressed radiologically died with 7 months of starting therapy.

### *Immunological assays*

These were performed on peripheral blood mononuclear cells (PBMC) isolated on Ficoll-Hypaque gradients from heparinised samples of peripheral venous blood obtained at the same time on each day. The natural killer (NK) cell activity of these PBMC was quantified by a standard 4 h $^{51}$Cr-release assay as previously described (Monson *et al.*, 1987) using the K562 erythroleukaemic cell line as an NK-sensitive target cell, or, for the quantification of spontaneous lymphocytotoxicity against an NK-resistant target, the colorectal cancer cell line, COLO205. Lymphokine-activated killer (LAK)

Correspondence: P.J. Guillou.
Received 24 April 1990; and in revised form 30 July 1990.

cells were also generated from these PBMC by a previously reported method (Guillou *et al.*, 1989). Briefly $10^7$ PBMC were cultured in 10 ml volumes of RPMI medium containing 10% fetal calf serum in the presence of 1,000 units $ml^{-1}$ of recombinant interleukin-2 (IL-2) (kindly provided by Euro-Cetus Ltd) for a period of 4 days. The activated lymphocytes recovered at the end of this period were then utilised as effector cells in a 4 h $^{51}Cr$-release assay against the NK-resistant DAUDI and COLO205 cell lines. On occasions when there were insufficient cells available to perform LAK assays against both targets, the cytotoxicity assay against COLO205 was omitted. NK and LAK assays were conducted at effector:target ratios ranging from 50:1 to 6.25:1. The percentage specific $^{51}Cr$ released at each effector:target cell ratio was calculated according to the usual formula as follows:

$$\% \text{ Specific } ^{51}\text{Cr released} = \frac{\text{experimental } ^{51}\text{Cr-release} - \text{spontaneous } ^{51}\text{Cr-release}}{\text{maximum } ^{51}\text{Cr-incorporated} - \text{spontaneous } ^{51}\text{Cr-release}} \times 100$$

where spontaneous $^{51}Cr$-release is that obtained from a 4 h incubation of $10^3$ target cells alone, and maximum $^{51}Cr$-release is that which is released following treatment of $10^3$ target cells with a detergent solution. In these studies the ratio of spontaneous to maximum $^{51}Cr$-release never exceeded 15%. The specific $^{51}Cr$-release data were then converted to lytic units defined as the number of cells per $10^7$ lymphocytes required to cause 30% specific $^{51}Cr$-release from the tumour target cell in question.

Unfractionated PBMC from these patients were also activated with concanavalin A (Con A) to quantify the effects of therapy on lymphocyte proliferation and IL-2 production. $10^5$ cells per well in 0.2 ml RPMI/10% FCS were cultured in quadruplicate in 96-well flat-bottomed microtitre plates (Nunc, Gibco Ltd, UK) in the presence of 10μg $ml^{-1}$ of Con A (Sigma, UK). Quadruplicate control wells containing no Con A were also prepared. The microplates were incubated for 24 h in 5% $CO_2$ in humidified air following which 100 μl supernatant was carefully withdrawn from each Con A activated well, pooled and stored at − 20°C for subsequent IL-2 assay. A similar volume was discarded from each of the unstimulated wells, and in all wells this was replaced by 100 μl medium containing 0.1 μCi of $^3H$-thymidine. Culture was continued for a further 16 h. The cells were harvested onto glass fibre discs using an automatic cell harvester (Dynatech, UK). The amount of $^3H$-thymidine incorporated was measured by beta counting and the data expressed as a stimulation index calculated as

$$SI = \frac{\text{mean c.p.m. in stimulated cultures}}{\text{mean c.p.m. in unstimulated cultures}}$$

*IL-2 assay*

The IL-2 content of supernatants from lectin-activated lymphocytes was measured by the bioassay described by Gillis *et al.* (1987) using the IL-2 dependent cloned murine CTLL-2 cell line as an indicator cell. These were cultured in 96-well flat bottomed microtitre trays at a concentration of $4 \times 10^3$ cells per well in the presence of doubling dilutions of supernatants derived from the Con A-activated peripheral blood mononuclear cells. The cultures were incubated for 24 h at 37°C in a humidified atmosphere of 5% $CO_2$ in air, pulsed with 0.1 μCi $^3H$-thymidine per well for 6 h, and then harvested for beta counting as described above. Each assay included doubling dilutions of a standard recombinant IL-2 preparation containing 100 Cetus units of IL-2 $ml^{-1}$ and the amount of IL-2 present in the supernatant in question was then calculated by probit analysis as previously described (Monson *et al.*, 1986).

*Statistical analyses*

For ease of presentation and analysis, data from the $^{51}Cr$-release assays was converted into $log_{10}$ lytic units. For the purposes of graphic presentation all lymphocytotoxicity data have been expressed as the mean ± the standard deviation of the mean $log_{10}$ lytic units. However, all statistical analyses were conducted using the non-parametric Mann–Whitney U test because even after logarithmic transformation the data were not parametric and it was not always feasible to perform every assay on every patient at every time point.

### Results

*Lymphocytotoxicity assays*

The changes occurring in NK activity during the course of treatment with 5-FU/folinic acid in these patients are shown in Figure 1. Note that the Y-axis is expressed as $log_{10}$ lytic units and so a relatively small apparent change represents a considerable actual change. Although there is a trend towards an increase in NK activity in the 4th and 5th weeks after the initiation of therapy compared with pre-treatment values, none of these changes was statistically significant. No spontaneous lymphocytotoxicity against the NK-resistant cell line COLO205 was observed.

In contrast, highly significant changes were observed in the capacity to generate LAK cells from the PBMC of these patients. The data from studies in which the international reference target for LAK cells, the DAUDI cell line was used as a LAK cell target are shown in Figure 2, where again the data are expressed in $log_{10}$ lytic units but on a rather more contracted vertical scale than those data shown in Figure 1. It can be seen that at the end of the first cycle of therapy (day 28) there was a statistically significant increase in the capacity to generate LAK cells from the peripheral blood mononuclear cells of these patients ($U = 109.5$, $P = 0.05$) and which was already evident on day 14 after the initiation of therapy ($U = 67$, $P = 0.015$). Furthermore, on measuring LAK generation 2 days following the administration of the next cycle of therapy (i.e. on day 35 following initiation of therapy) there was a further rise in LAK generation which was again significantly greater than that seen prior to treatment ($U = 8$, $P = 0.005$) and also significantly higher than that seen at the end of the first month of therapy on day 28 ($U = 19$, $P = 0.049$). Thereafter, LAK cell generation progressively declined although on day 56 of the treatment it still remained significantly higher than that seen on day 0 ($U = 50$, $P = 0.036$). By the 12th week after initiation of treatment, LAK cell generation was not significantly different from that seen prior to treatment ($U = 20$, $P = 0.14$), although the numbers of patients available for study at this stage were smaller, 12 patients having been withdrawn from therapy.

A similar trend was observed when the COLO205 cell line was employed as a LAK cell target (Figure 3). Compared with pre-treatment figures, LAK cell generation was significantly higher on days 14 ($U = 74$, $P = 0.029$), 28

**Figure 1** Changes observed in Natural killer cell activity during chemotherapy with 5-FU/folinic acid. Data are expressed as mean log lytic units ± s.d. Bold horizontal arrows denote periods of infusion of 5-FU/folinic acid.

**Figure 2** Changes observed in the capacity to generate Lymphokine-activated killer cells from the peripheral blood mononuclear cells of patients with colorectal cancer during therapy with 5-FU/folinic acid. Target cell, DAUDI. Otherwise legend as for Figure 1.

**Figure 3** Legend as for Figure 2 except that the data shown are those obtained using the COLO205 cell line as a target in the LAK cell assay.

(U = 104.5, $P = 0.024$) and 56 (U = 34.5, $P = 0.012$), although by day 84 the apparently higher LAK cell generation seen in these patients at this point did not reach statistical significance (U = 16.5, $P = 0.077$).

The mean $\log_{10}$ LAK activity induced against DAUDI cells at 1 and 2 months of therapy in patients who either had responded or had stable disease ($n = 9$) was higher than that of lymphocytes obtained from those with progressive disease ($n = 12$), although this did not achieve statistical significance (3.33 ± 1.2 *vs* 2.58 ± 0.79, $P = 0.18$ at 28 days and 3.14 ± 1.07 *vs* 2.35 ± 0.19, $P = 0.08$ at 56 days). A similar trend was seen when LAK activity against the COLO205 cell line was compared at these two time points (2.7 ± 0.16 *vs* 2.33 ± 0.52, $P = 0.18$ at 28 days and 3.5 ± 0.9 *vs* 2.4 ± 0.65, $P = 0.08$ at 56 days).

*Lectin-induced lymphocyte proliferation*

The pattern of change in lectin (Con A)-induced lymphocyte proliferation during this therapy are shown in Figure 4. No significant changes were observed until the 28th day after the initiation of treatment (i.e. just prior to the administration of the second cycle of 5-FU/folinic acid), when the mean stimulation index was significantly higher than that observed prior to therapy (U = 78, $P = 0.042$). The stimulation index then appeared to fall immediately following the second cycle of treatment although it did not descend below baseline values. Thereafter the mean stimulation index rose progressively and just prior to the third infusion cycle was again significantly higher than that seen prior to therapy (U = 18, $P = 0.02$) and also was significantly higher than that seen on day 35 (U = 25, $P = 0.04$). Data obtained subsequent to day 56 of treatment were too small for statistical analysis to be valid.

*IL-2 production*

The production of IL-2 by Con-A-activated lymphocytes during 5-FU/folinic acid therapy is shown in Figure 5. There

**Figure 4** Changes in lectin (Con A)-induced lymphocyte proliferation in patients with colorectal cancer during chemotherapy with 5-FU/folinic acid. Data expressed as mean stimulation index ± s.d.

**Figure 5** Interleukin-2 production by lectin-activated lymphocytes from patients with colorectal cancer during chemotherapy with 5-FU/folinic acid. Data are expressed as mean IL-2 production ± s.d.

was no diminution of IL-2 production at any stage throughout the period of treatment although the numbers of available lymphocytes for these and all the other assays became attenuated during the second and third cycles of therapy. Nevertheless, by day 28 the amount of biologically active IL-2 in the supernatants of Con A-activated peripheral blood mononuclear cells from these patients was significantly higher than that measured under the same conditions prior to the initiation of therapy (U = 24, $P = 0.046$). By day 56 the supernatant IL-2 content was not significantly different from that of supernatants from cells obtained prior to treatment (U = 18, $P = 0.093$). Again, data obtained beyond day 56 of the treatment cycle was inadequate for statistical analysis.

*Lymphocyte:monocyte ratios before and during treatment*

The mean lymphocyte:monocyte ratio in the peripheral blood before the initiation of therapy was 2.65:1. At 28 days of therapy it was 2.26:1 and after 56 days of treatment it was 2.5:1. Neither of these ratios was significantly different from that observed prior to treatment.

### Discussion

We were motivated to conduct this investigation because of the current vogue for the design of therapeutic protocols which combine chemotherapy with IL-2 for the treatment of refractory solid tumours. Some of these protocols, such as those which involve pre-treatment with cyclophosphamide (CY), have undergone extensive pre-clinical study and have a sound scientific basis. Thus, CY attenuates the toxicity of IL-2 in mice, and exerts a synergistic therapeutic effect with

IL-2 on relatively IL-2 resistant tumours in rodents (Papa *et al.*, 1988; Rosenstein *et al.*, 1986). This combination may have some merit when applied clinically (Mitchell *et al.*, 1988).

However, other protocols have been introduced on an empirical basis without prior study of the influence which the chemotherapeutic agent has on the desirable immunological events induced by IL-2 (Stoter *et al.*, 1989; Flaherty, 1989). Before embarking on the design of a chemo-immunotherapeutic protocol which involved the combination of IL-2 with 5-FU in patients with colorectal cancer, we wished to determine the influence on IL-2-based responses of a 5-FU regimen with demonstrable therapeutic efficacy. Currently this is considered to be best achieved by combining 5-FU with folinic acid (Erlichman *et al.*, 1988; Kerr, 1989). We anticipated that lymphocyte responses to cytokine activation would be diminished following an infusion of folinic acid accompanied by intravenous bolus 5-FU for 5 days. However, this combination proved not to be immunosuppressive and was followed by an augmented capacity to generate LAK cells from the peripheral blood lymphocytes of patients within the first month of initiating treatment. After the first month of therapy, wide individual variations in the capacity to generate LAK cells from the lymphocytes of these patients were observed but the augmented responses were maintained through second and third cycles of treatment although the magnitude of the subsequent changes appeared less marked than those observed through the first cycle of treatment (Figure 2).

In considering the mechanism of this augmented LAK induction following 5-FU/folinic acid treatment it is important to note that this occurred without any significant increase of similar magnitude in NK activity. Classical NK cells with the $CD3^-$, $CD16^+$, $CD56^+$ phenotype are considered to represent the principal precursor lymphocyte population from which LAK cells are derived on exposure to IL-2 (Herberman *et al.*, 1987). These data would therefore suggest that the capacity for augmented LAK cell induction seen following this treatment is due to an increase in the sensitivity of LAK precursors to activation with IL-2 rather than to an increase in the circulating NK-derived precursor pool. In this context our data bear some similarities with those described by Arinaga *et al.* (1986) following a single intravenous dose of Adriamycin, and Kiyohara *et al.* (1988) during intensive polychemotherapy for urinary tract cancer. However, NK cells are not the sole source of precursors for the generation of non-MHC-restricted lymphocytotoxicity. For example, $CD3^+$ T-cells which possess the $\gamma/\delta$ T-cell receptor have been found to exert non-restricted tumour cell lysis on exposure to IL-2 *in vitro* (Borst *et al.*, 1987) and expansion of this small component of peripheral blood $CD3^+$ lymphocytes cannot be excluded from the present results. We are currently conducting flow cytometric studies to determine what, if any, phenotypic changes underlie these observations because of the different receptor-ligand interactions which direct allospecific and NK-associated cellular cytotoxicity exerted by T-cells (Koide *et al.*, 1989).

The immunological changes which occur following intravenously administered 5-FU/folinic acid do not appear to be limited to MHC-unrestricted cellular cytotoxicity. In these preliminary studies we have also found lectin-induced lymphocyte proliferation and IL-2 released from these activated T-cells to be increased, at least within the first month of therapy. Thereafter the changes are rather more erratic and difficult to interpret, possibly because fewer patients were available for study as patients with progressive disease were withdrawn from treatment. Nevertheless, these results suggest that the effects of this 5-FU/folinic acid regimen on the immune system may be generalised and not simply restricted to lymphocytotoxic precursors although we have not measured LAK precursor activity in patients after cessation of therapy to determine whether LAK induction fell to pretreatment values.

Accepting the difficulties inherent in comparing cytotoxicity data from one laboratory to another, the magnitude of change in LAK precursor availability we have observed following 5-FU/folinic acid therapy is at least comparable to that reported during the immediate 1 to 2 days of rebound lymphocytosis following therapy with IL-2 alone. It has been suggested that the magnitude of this rebound lymphocytosis, though not the level of inducible cytotoxicity, correlates with clinical response (West *et al.*, 1987; Boldt *et al.*, 1988). Others have suggested that a high level of IL-2-primed LAK precursors occurs during the post-IL-2 rebound and is responsible for the response if further IL-2 is administered (Schoof *et al.*, 1988). Whatever the mechanism of the changes we describe, the clinical implications of these data are that at the very worst this therapeutic combination does not impair the development of the cellular cytotoxic responses which underlie IL-2 therapy and may promote them. On the basis of the results obtained from this study, such a regimen should administer 5-FU/folinic acid first, followed by IL-2 14 days later when there is an enhanced capacity for circulating LAK cell precursors to generate a cytolytic response. These data therefore provide a logical basis upon which to combine these two different therapeutic approaches in the hope of increasing clinical response rates in the treatment of unresectable colorectal cancer.

### References

ADES, E.W., MCKEMIE, C.R., WRIGHT, S., PEACOCKE, N., PANTAZIS, C. & LOCKHART, W.L. (1987). Chemotherapy subsequent to recombinant interleukin-2 immunotherapy: Protocol for enhanced tumoricidal activity. *Nat. Immunol. Cell Growth Regul.*, **6**, 260.

ARBUCK, S.G. (1989). Overview of clinical trials using 5-FU and Leucovorin for the treatment of colorectal cancer. *Cancer*, **63**, 1036.

ARINAGA, S., AKIYOSHI, T. & TSUJI, H. (1986). Augmentation of the generation of cell-mediated cytotoxicity after a single dose of Adriamycin in cancer patients. *Cancer Res.*, **46**, 4213.

BOLDT, D.H., MILLS, B.J., GEMLO, B.T. & 11 others (1988). Laboratory correlates of adoptive immunotherapy with recombinant Interleukin-2 and lymphokine-activated killer cells in humans. *Cancer Res.*, **48**, 4409.

BORST, J., VAN DE GRIEND, J.W., VAN OOSTVEEN, S.-L. & 4 others (1987). A T-cell receptor/CD3 complex found on cloned functional lymphocytes. *Nature*, **325**, 683.

DOROSHOW, J.H., BERTRAND, M., NEWMAN, E. & 8 others (1987). Preliminary analysis of a randomised comparison of 5-Fluorouracil versus 5-Fluorouracil and high dose continuous infusion folinic acid in disseminated colorectal cancer. *NCI Monogr.*, **5**, 171.

EGGERMONT, A.M.M. & SUGARBAKER, P.H. (1988). Efficacy of chemoimmunotherapy with cyclophosphamide, interleukin-2 and lymphokine-activated killer cells in an intraperitoneal murine tumour model. *Br. J. Cancer*, **58**, 410.

ERLICHMAN, C., FINE, S., WONG, A. & ELHAKIM, T. (1988). A randomised trial of Fluorouracil and folinic acid in patients with metastatic colorectal carcinoma. *J. Clin. Oncol.*, **6**, 469.

FLAHERTY, L. (1989). The combination of recombinant interleukin-2 and dacarbazine (DTIC) in metastatic malignant melanoma. *Cancer Treatment Rev.*, **16** (suppl. A), 65.

GILLIS, S., FERM, M.M., OU, W. & SMITH, K.A. (1978). T-cell growth factor: parameters of production and a quantitative microassay for activity. *J. Immunol.*, **120**, 2027.

GUILLOU, P.J., SEDMAN, P.C. & RAMSDEN,C.W. (1989). Inhibition of lymphokine-activated killer cell generation by cultured tumor cell lines *in vitro*. *Cancer Immunol. Immunother.*, **28**, 43.

HAMBLIN, T.J., INZANI, V., SADULLAH, S. & 5 others (1989). A phase II trial of recombinant interleukin-2 and 5-FU chemotherapy in patients with metastatic colorectal carcinoma. *Cancer Treatment Rev.*, **16** (suppl. A), 163.

HERBERMAN, R.B., HISERODT, J., VUJANOVIC, N. & 11 others (1987). Lymphokine-activated killer cell activity. Characteristics of effector cells and their progenitors in blood and spleen. *Immunol. Today*, **8**, 178.

KEMPF, R.A. & MITCHELL, M.S. (1984). Effects of chemotherapeutic agents on the immune response. *Cancer Invest.*, **2**, 459.

KERR, D.J. (1989). 5-Fluorouracil and folinic acid: interesting biochemistry or effective treatment? *Br. J. Cancer*, **60**, 807.

KIYOHARA, T., TANIGUCHI, K., KUBOTA, S., KOGA, S., SAKURAKI, T. & SAIITOH, Y. (1988). Induction of lymphokine-activated killer-like cells by cancer chemotherapy. *J. Exp. Med.*, **168**, 2355.

KOIDE, J., RIVAS, A. & ENGELMAN, E.G. (1989). Natural killer (NK)-like cytotoxic activity of allospecific T cell receptor-$\gamma$, $\delta^+$ T cell clones. *J. Immunol.*, **142**, 4161.

LINDEMANN, A., HOEFFKEN, K., SCHMIDT, R.E. & 9 others (1989). A multicenter trial of interleukin-2 and low-dose cyclophosphamide in highly chemotherapy-resistant malignancies. *Cancer Treatment Rev.*, **16** (suppl. A), 53.

MITCHELL, M.M., KEMPF, R.A., HAREL, W. & 4 others (1988). Effectiveness and tolerability of low-dose cyclophosphamide and low-dose intravenous interleukin-2 in disseminated melanoma. *J. Clin. Oncol.*, **6**, 409.

MONSON, J.R.T., RAMSDEN, C.W. & GUILLOU, P.J. (1986). Decreased interleukin-2 production in patients with gastrointestinal cancer. *Br. J. Surg.*, **73**, 483.

MONSON, J.R.T., RAMSDEN, C.W., GILES, G.R., BRENNAN, T.G. & GUILLOU, P.J. (1987). Lymphokine-activated killer (LAK) cells in patients with gastrointestinal cancer. *Gut*, **28**, 1420.

NORTH, R.J. (1982). Cyclophosphamide-facilitated adoptive immunotherapy of an established tumor depends on elimination of tumor induced suppressor T cells. *J. Exp. Med.*, **155**, 1063.

PAPA, M.Z., VETTO, J.T., ETTINGHAUSEN, S.E., MULE, J.J. & ROSENBERG, S.A. (1986). Effects of corticosteroids on the antitumor activity of lymphokine-activated killer cells and interleukin 2 in mice. *Cancer Res.*, **46**, 5618.

PAPA, M.Z., YANG, J.C., VETTO, J.T., SHILONI, E., EISENTHAL, A. & ROSENBERG, S.A. (1988). Combined effects of chemotherapy and interleukin 2 in the therapy of mice with advanced pulmonary tumors. *Cancer Res.*, **48**, 122.

POWELL, C.B., MUTCH, D.G., KAO, M.S. & COLLINS, J.L. (1990). Reduced natural cytotoxic cell activity in patients receiving cisplatin-based chemotherapy and in mice treated with cisplatin. *Clin. Exp. Immunol.*, **79**, 424.

ROSENBERG, S.A., LOTZE, M.T., YANG, J.C. & 4 others (1989). Experience with the use of high dose interleukin 2 in the treatment of 652 cancer patients. *Ann. Surg.*, **214**, 474.

ROSENSTEIN, M., ETTINGHAUSEN, S.E. & ROSENBERG, S.A. (1986). Extravasation of intravascular fluid by the systemic administration of recombinant interleukin 2. *J. Immunol.*, **137**, 1735.

SCHOOF, D.D., GRAMOLINI, B.A., DAVIDSON, D.D., MASSARO, A.F., WILSON, R.E. & EBERLEIN, T.J. (1988). Adoptive immunotherapy of human cancer using low-dose recombinant interleukin 2 and lymphokine-activated killer cells. *Cancer Res.*, **48**, 5007.

STOTER, G., SHILONI, E., GUNDERSEN, S. & 7 others (1989). Alternating recombinant human interleukin 2 and dacarbazine in advanced melanoma. A multicentric phase II study. *Cancer Treatment Rev.*, **16** (suppl. A), 59.

VETTO, J.T., PAPA, M.Z., LOTZE, M.T., CHANG, A.E. & ROSENBERG, S.A. (1987). Reduction of toxicity of IL-2 and LAK cells in humans by the administration of corticosteroids. *J. Clin. Oncol.*, **5**, 496.

WEST, W.H., TAUER, K.W., YANELLI, J.R. & 4 others (1987). Constant-infusion recombinant interleukin-2 in adoptive immunotherapy of advanced cancer. *N. Engl. J. Med.*, **316**, 898.